# Ethical dimensions of healthcare nudges: a PRISMA-ScR–guided scoping review and framework for responsible behavioral governance

**DOI:** 10.3389/fpubh.2025.1716466

**Published:** 2025-11-26

**Authors:** Huijing Shang, Lingfang Xiong, Kefan Chen, Renqiong Tian, Jing Tu, Xianhui Shang

**Affiliations:** 1 Department of Rheumatology and Immunology, The First People’s Hospital of Zunyi (The Third Affiliated Hospital of Zunyi Medical University), Zunyi, China; 2 Department of Rheumatology and Immunology, The Second Affiliated Hospital of Guizhou University of Traditional Chinese Medicine, Guiyang, China; 3 Department of Pediatric Rehabilitation, The First People’s Hospital of Zunyi (The Third Affiliated Hospital of Zunyi Medical University), Zunyi, China; 4 Department of Pediatric Surgery, Affiliated Hospital of Zunyi Medical University, Zunyi, China; 5 Department of Pediatric Surgery, Guizhou Children’s Hospital, Zunyi, China

**Keywords:** nudge theory, healthcare ethics, PRISMA-ScR, autonomy, behavioral governance, bioethics, health policy ethics, behavioral economics

## Abstract

**Background:**

Nudging has gained momentum in healthcare as a behavioral strategy to support beneficial health choices. However, its ethical legitimacy remains contested, especially regarding autonomy, transparency, fairness, and risks of covert influence.

**Objective:**

This scoping review synthesizes ethical debates on healthcare nudges and proposes governance principles to guide ethically responsible implementation.

**Methods:**

We conducted a scoping review following the PRISMA-ScR framework across international and Chinese databases. Conceptual and empirical bioethics literature from 2013 to 2023 was analyzed through thematic synthesis.

**Results:**

Twenty-three studies met inclusion criteria. Ethical concerns centered on autonomy (22/23), informed consent and transparency (4/23), equity and distributive fairness (2/23), and risk of unintended harm (1/23). Reflective nudges that support deliberation were more widely endorsed than covert defaults. Recent scholarship emphasizes transparency, proportionality, stakeholder participation, and equity audits as conditions for ethical legitimacy.

**Contribution:**

To our knowledge, this is the first review to systematically synthesize ethical dimensions of healthcare nudges using the PRISMA-ScR framework and to propose an actionable governance model for responsible behavioral regulation. The review advances ethical theory by integrating principlism, consequentialism, and deontology with cross-cultural public health ethics, and policy insights by identifying operational safeguards for health systems.

**Conclusion:**

Healthcare nudges can be ethically justified when designed with transparency, meaningful opt-out options, proportionality of influence, and equity safeguards. Responsible behavioral governance requires culturally sensitive implementation, independent oversight, and continuous monitoring of autonomy and fairness outcomes. Future empirical work should examine real-world impacts on patient agency and equity across diverse clinical and cultural contexts.

## 1 Background

Nudge theory—originally conceptualized by Thaler and Sunstein as a form of “libertarian paternalism” that shapes choices without restricting options—has become increasingly influential in healthcare policy and clinical practice (1, 2). Nudges have been deployed to improve vaccination uptake, chronic disease prevention, organ donation, medication adherence, and digital health engagement.

Despite their promise, nudges have sparked vigorous ethical debate. A central fault line exists between Sunstein’s defense of nudges as autonomy-preserving tools that steer individuals toward welfare-enhancing behaviors and Hausman’s critique that nudges may disguise coercion under the guise of freedom, thereby compromising authentic self-determination (3, 4). This debate anchors broader philosophical disputes between:

1. liberal paternalism vs. autonomy maximization,
2. welfare promotion vs. manipulation avoidance, and
3. subtle influence vs. explicit consent in health decision-making.

Autonomy, informed consent, and public health ethics are conceptually interconnected in these debates. Autonomy requires individuals to understand influences on their choices and retain voluntary control. Informed consent operationalizes autonomy by demanding transparency and comprehension. Public health ethics balances individual freedom with collective welfare, prompting ongoing tension between voluntariness and utilitarian justification in behavioral interventions (2, 5).

While nudging has often been ethically defended on consequentialist or utilitarian grounds—particularly where it reduces disease burden or enhances health equity—critics argue that hidden steering may bypass deliberation and violate procedural respect for persons, even when outcomes are beneficial (4, 6). Recent scholarship advances frameworks such as ethical behavioral governance and participatory nudging to reconcile these tensions by emphasizing transparency, stakeholder engagement, and accountability (7–9).

Furthermore, cultural values shape ethical interpretations of nudging. Western liberal bioethics prioritizes individual autonomy, explicit consent, and freedom from interference, making transparency a prerequisite for ethical legitimacy (10). In contrast, East Asian contexts rooted in Confucian and communitarian philosophies place greater emphasis on relational autonomy, social harmony, and solidarity, making public-health nudges more acceptable when aligned with collective welfare and culturally situated norms (11, 12). Yet, critics warn that invoking collective good must not legitimize unchecked paternalism, reinforcing the need for culturally sensitive but principled safeguards.

Given these contested ethical landscapes, a scoping review design is well-suited to map evolving arguments, identify conceptual patterns, and synthesize multidisciplinary discourse across bioethics, public health, behavioral economics, and political philosophy. Unlike narrative reviews, scoping reviews allow systematic identification of competing ethical positions and emergent trends, which is essential when navigating morally charged, rapidly developing policy tools such as nudges.

## 2 Methods

This scoping review was conducted in accordance with the PRISMA-ScR (Preferred Reporting Items for Systematic Reviews and Meta-Analyses extension for Scoping Reviews) framework to ensure methodological transparency and reproducibility. The review aimed to address two primary research questions: (1) What barriers hinder the resolution of ethical concerns associated with applying nudge theory in healthcare? and (2) What strategies have been proposed to manage these ethical issues?

### 2.1 Search strategy

A comprehensive literature search was performed across both international and Chinese databases, including the Cochrane Library, CINAHL, Embase, PubMed, Web of Science, China National Knowledge Infrastructure (CNKI), VIP Database, Wanfang Data, and SinoMed (CBMdisc). All databases were searched from their inception to December 31, 2024, with no restrictions on study design but limited to English and Chinese publications.

The search strategy combined controlled vocabulary and free-text terms across three main domains: (1) “nudge,” “nudging,” or “nudge theory”; (2) “health,” “medical,” “clinical,” or “public health”; and (3) “ethic,” “ethics,” or “ethical issue.” Boolean operators (AND/OR) were used to structure the queries.

An example Boolean search string (PubMed) is as follows:

(“nudge” OR “nudging” OR “nudge theory”) AND (“health” OR “medicine” OR “public health” OR “clinical practice”) AND (“ethics” OR “ethical issue” OR “bioethics”).

### 2.2 Eligibility criteria

Eligibility was defined using the PCC (Population–Concept–Context) framework (13). Studies were included if they:

(P) Discussed patients, healthcare providers, or public populations affected by nudges;
(C) Examined ethical issues related to nudge theory (e.g., autonomy, consent, justice, risk); and.
(C) Were situated in health-related domains such as clinical care, disease prevention, nutrition, pharmacotherapy, digital health, or public health policy.

Exclusion criteria were: (1) non-English or non-Chinese publications; (2) absence of full-text access; and (3) articles that did not explicitly address ethical issues related to nudge applications.

### 2.3 Study selection

The study selection process followed PRISMA 2020 recommendations. All retrieved records were imported into NoteExpress (version 3.9) for duplicate removal. Two independent reviewers screened titles and abstracts against the inclusion criteria. Disagreements were resolved through discussion, and if consensus could not be reached, a third reviewer adjudicated the decision. To ensure inter-rater reliability, a pilot calibration exercise was conducted prior to full screening, resulting in a Cohen’s kappa coefficient of 0.87, indicating strong agreement.

**Figure 1:**
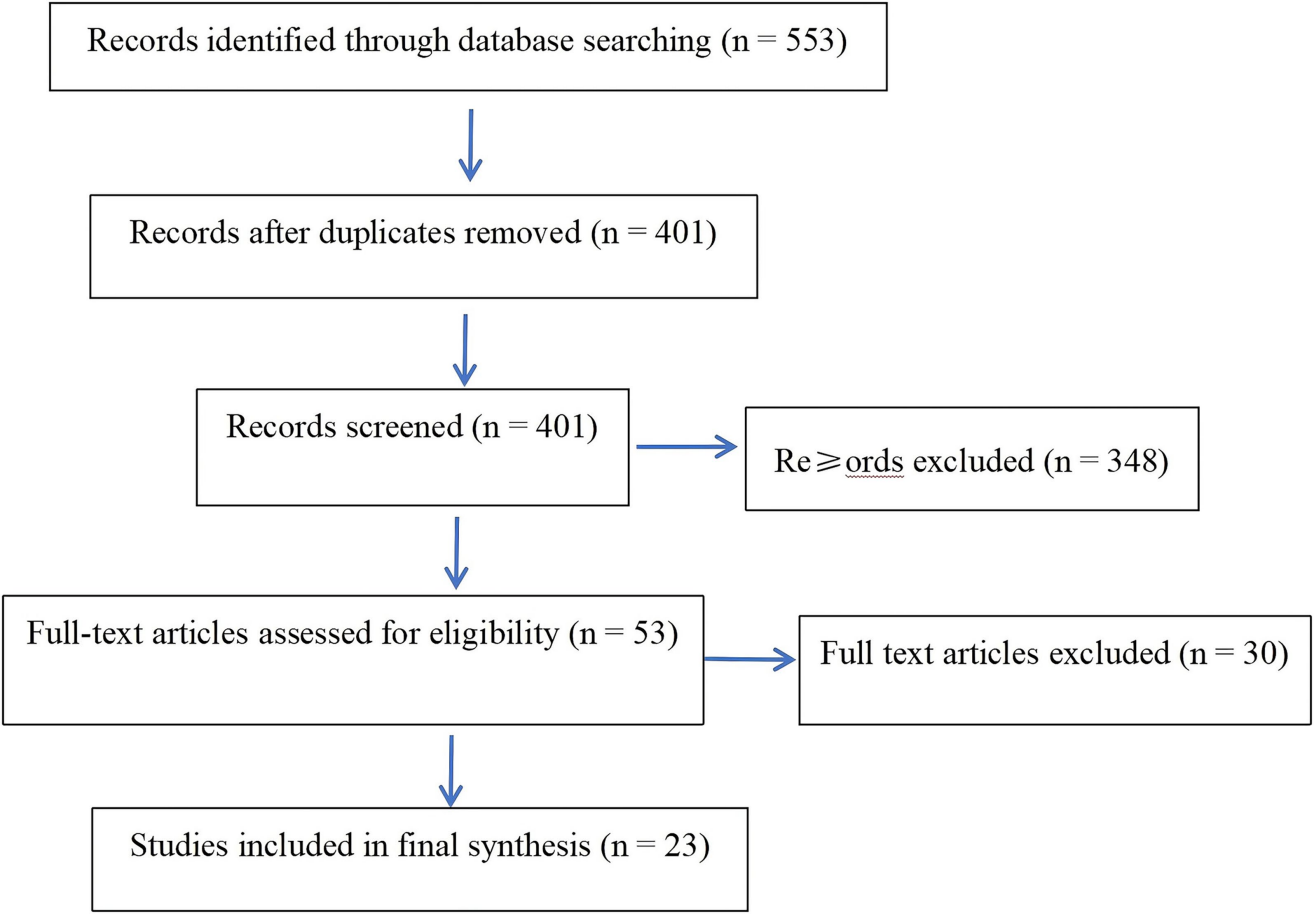
PRISMA 2020 flow diagram for study selection.

A total of 553 records were identified. After removing duplicates, 401 unique studies remained. Following title and abstract screening, 53 full-text articles were assessed for eligibility. Thirty were excluded for not meeting inclusion criteria, leaving 23 studies for final synthesis (6, 8, 14–34). The complete selection process is depicted in Figure 1 (PRISMA Flow Diagram).

### 2.4 Data extraction and synthesis

Data extraction was independently performed by two reviewers using a standardized data charting form developed for this study. Extracted variables included:

- author(s) and year of publication,
- country or region,
- study design,
- ethical domains addressed, and.
- proposed coping strategies or ethical frameworks.

To ensure consistency, reviewers underwent initial calibration training and pilot testing of the extraction form. All disagreements during data charting were discussed and resolved collaboratively, with audit verification by a senior reviewer.

The extracted data were summarized using descriptive statistics and thematic synthesis. Frequency analysis was applied to identify the most prevalent ethical issues (e.g., autonomy, consent, fairness, risk). Qualitative findings were organized into four major ethical domains—autonomy, informed consent, distributive justice, and risk mitigation—to facilitate comparison across studies.

### 2.5 Detailed search strategy

To ensure transparency and reproducibility, the complete Boolean search strings for all databases are provided below, in accordance with PRISMA-ScR guidelines.PubMed(“nudge” OR “nudging” OR “nudge theory”)
AND (“health” OR “medicine” OR “public health” OR “clinical practice”)
AND (“ethics” OR “ethical issue” OR “bioethics”)*Date range*: From inception to December 31, 2024*Language restriction*: English OR Chinese
Embase
(‘nudge theory’/exp. OR ‘nudge’:ti,ab,kw OR ‘nudging’:ti,ab,kw)
AND (‘health care’/exp. OR ‘public health’/exp. OR ‘medicine’:ti,ab,kw).
AND (‘ethics’/exp. OR ‘ethical issue’:ti,ab,kw).*Language restriction*: English OR Chinese
Web of Science
TS = ((“nudge” OR “nudging” OR “nudge theory”)
AND (“health” OR “medicine” OR “public health”)
AND (“ethics” OR “ethical issue” OR “bioethics”))*Date range*: Up to December 31, 2024
Cochrane Library
(“nudge” OR “nudging” OR “nudge theory”)
AND (“ethics” OR “bioethics” OR “ethical issue”)*Scope*: Reviews, protocols, and trials relevant to ethical design in healthcare
CINAHL
(MH “Nudge Theory” OR TI “nudge” OR AB “nudging”)
AND (MH “Health Behavior” OR “public health” OR “clinical practice”)
AND (MH “Ethics” OR “ethical issue” OR “bioethics”)*Filters applied*: Peer-reviewed articles; English or Chinese
CNKI (China National Knowledge Infrastructure)
Subject = (Nudge OR Nudge Theory)
AND Subject = (Ethics OR Ethical Issue OR Medical Ethics)*Date range*: From inception to December 31, 2024
Wanfang Database
Subject = (Nudge OR Nudge Theory)
AND (Medicine OR Public Health OR Health Management)
AND (Ethics OR Ethical Issue)
VIP Database
(“Nudge” OR “Nudge Theory”) AND (“Ethics” OR “Ethical Issue”) AND (“Health” OR “Medicine”)
SinoMed (CBMdisc)
(“Nudge” OR “Nudge Theory”) AND (“Ethics” OR “Ethical Issue”) AND (“Healthcare” OR “Public Health”)


Notes

- The search cutoff date for all databases was December 31, 2024.
- Only English and Chinese studies were included.
- All searches were independently conducted and verified by two reviewers, with third-party validation.
·All records were imported into NoteExpress (v3.9) for deduplication and management.
- The detailed selection and screening process is illustrated in Figure 1 (PRISMA Flow Diagram).

### 2.6 Data analysis

Extracted data were synthesized using a qualitative thematic approach. Following iterative review and coding, ethical issues were categorized into conceptual domains (autonomy, informed consent, distributive justice, and risk). Frequency counts were used to summarize the prevalence of each domain across included studies. No meta-analysis was conducted, consistent with the methodological nature of a scoping review. All analysis procedures adhered to PRISMA-ScR guidance to ensure rigor, transparency, and reproducibility.

## 3 Results

### 3.1 Study selection

A total of 553 records were identified through database searches. After removing 152 duplicates, 401 unique records remained for screening. Following title and abstract screening, 348 articles were excluded for not meeting eligibility criteria. Fifty-three full-text articles were reviewed in detail, resulting in 30 exclusions due to lack of ethical focus or insufficient relevance to healthcare nudging. Ultimately, 23 studies were included in the final synthesis (Figure 1).

### 3.2 Characteristics of included studies

The 23 included studies were published between 2013 and 2023. Most originated from high-income Western countries including the United States (*n* = 8) (12, 13, 15, 19, 21, 24–26), Norway (*n* = 3) (14, 18, 29), the United Kingdom (*n* = 2) (16, 23), and the Netherlands (*n* = 2) (20, 28). A smaller but meaningful proportion came from Asian regions such as China and Singapore (5, 8), as well as additional countries including France (30), Greece (11), Italy (22), Israel (27), Denmark (17), and Brazil (6), reflecting a growing global interest in ethical dimensions of nudging.

Most studies employed conceptual or normative ethical analysis, while several incorporated case-based ethical reflection or policy evaluation (Table 1).

**Table 1:** Characteristics of studies included in the scoping review (*n* = 23).

Study	Country/Region	Study design	Healthcare context
Study 1	USA	Conceptual Ethics Analysis	Vaccination Policy
Study 2	UK	Policy Ethics Review	Organ Donation
Study 3	Norway	Normative Ethical Analysis	Chronic Disease Prevention
Study 4	China	Conceptual Discussion	Public Health Campaigns
Study 5	Singapore	Case-based Ethical Analysis	Digital Health Nudges
Study 6	USA	Ethical Commentary	Smoking Cessation
Study 7	USA	Conceptual Framework Analysis	Childhood Obesity Prevention
Study 8	Netherlands	Normative Bioethics	End-of-Life Care
Study 9	Norway	Policy Ethics Analysis	Alcohol Consumption Campaign
Study 10	Australia	Conceptual Policy Ethics	HPV Vaccination
Study 11	UK	Ethical Review	Nutrition Labeling
Study 12	USA	Legal-Ethical Analysis	Sugary Drink Tax Policy
Study 13	Canada	Conceptual Bioethics	Mental Health Services
Study 14	Germany	Ethical Theoretical Study	Hospital Quality Indicators
Study 15	Denmark	Normative Analysis	General Preventive Medicine
Study 16	Netherlands	Case-based Ethics	Hospital Infection Control
Study 17	Norway	Conceptual Ethics	Cancer Screening
Study 18	Sweden	Ethical Commentary	Antibiotic Stewardship
Study 19	Japan	Conceptual Discussion	Health Technology Adoption
Study 20	Singapore	Policy Evaluation	COVID-19 Digital Tracing
Study 21	China	Ethical Framework Study	Vaccination Hesitancy
Study 22	USA	Normative Bioethics	Organ Donation Consent System
Study 23	Italy	Ethical Analysis	Lifestyle-related Diseases

Most studies adopted conceptual or normative ethical frameworks and addressed autonomy and transparency across diverse healthcare settings.

### 3.3 Ethical themes identified

Across the included studies, four core ethical domains emerged (Table 2). Autonomy-related concerns were the most prevalent, appearing in 22 studies (5, 6, 8, 11–23, 25–30), highlighting risks of covert manipulation, coercive defaults, and diminished voluntariness. Issues of informed consent and transparency were discussed in four studies (13, 14, 19, 24), focusing on inadequate disclosure and limited user understanding. Equity and distributive fairness concerns appeared in two studies (5, 12), emphasizing disproportionate effects on vulnerable populations. One study (18) identified risk of harm, noting unintended negative outcomes associated with nudging strategies.

**Table 2:** Ethical domains and frequency across studies.

Ethical Domain	Description	Frequency (n)	%	Example Concerns
Autonomy	Freedom of choice, voluntariness, avoidance of manipulation	**22**	**95.7%**	Covert influence, reduced agency
Informed Consent & Transparency	Clear and truthful communication of nudges	**4**	**17.4%**	Opacity of nudging mechanisms
Equity & Fairness	Avoiding disproportionate impact on vulnerable groups	**2**	**8.7%**	Socioeconomic disparities
Risk of Harm	Preventing unintended negative outcomes	**1**	**4.3%**	Behavioral over-dependence

Autonomy was the dominant ethical concern across nearly all studies, followed by transparency-related consent issues. Bold values indicate the highest frequency observed among the ethical domains.

### 3.4 Synthesis of key trends

Across included studies, autonomy and transparency emerged as the central normative benchmarks in ethical evaluation of nudges. Evidence suggests that ethically acceptable nudges should be:

- Clear and transparent.
- Respectful of individual deliberation.
- Proportionate in behavioral influence.
- Aligned with long-term welfare.

Narrative comparisons showed a general preference for Type 2 (reflective) nudges over Type 1 (automatic) nudges, particularly in clinical contexts where patient agency must remain paramount.

Emerging research—especially in the context of digital health and algorithmic guidance—raised concerns regarding opacity, hidden persuasion, and unequal behavioral effects, underscoring the need for procedural fairness and stakeholder engagement.

## 4 Discussion

This review demonstrates that ethical debate around healthcare nudges remains dominated by concerns about autonomy and transparency, accompanied by increasing attention to equity and distributive justice. Across empirical and normative literatures, respect for individual agency, intelligible disclosure, proportionality of influence, and fairness in behavioral impact emerged as core ethical benchmarks. Reflective nudges that promote deliberation were more widely endorsed than automatic nudges that subtly manipulate default behaviors, particularly in clinical and consent contexts. Recent research suggests that algorithmically mediated nudges may intensify these concerns by introducing opaque cognitive steering mechanisms, which necessitate new transparency protocols and governance safeguards (35).

The integration of principlism, consequentialism, and deontology provides a coherent ethical foundation for evaluating nudging interventions. From a principlist lens, protecting autonomy and avoiding harm remain essential conditions for ethical legitimacy (31). Consequentialist reasoning supports nudges when measurable public health benefits outweigh minimal intrusion, provided transparency safeguards are upheld (33, 36). By contrast, a deontological perspective demands truthful and non-coercive influence regardless of outcome, a position that strengthens ethical requirements for disclosure and voluntariness (37). Emerging theoretical models such as “boosting” or “self-nudging” prioritize individual agency and cognitive empowerment over external control, aligning with democratic and participatory frameworks for behavioral policy (38).

Recent scholarship emphasizes “responsible behavioral governance” models, including public justification of nudging mechanisms, participatory design, and equity audits in digital health contexts (36, 39, 40). These developments reflect a shift from libertarian paternalism to procedural ethics, in which legitimacy depends not only on outcomes but also on transparency, accountability, and cultural sensitivity. Notably, scholars have raised concerns that predictive governance systems—especially those embedded within AI infrastructures—may erode voluntariness and blur the line between influence and manipulation, prompting calls for algorithmic accountability and ethical constraints (41).

In East Asian contexts informed by collectivist ethics, nudges aimed at promoting shared welfare may obtain broader acceptance when transparency and community benefit are clearly communicated (12, 42). However, recent psychological analysis indicates that AI-enabled nudging may also trigger unintended mental health effects, such as reduced agency or perceptual dissonance, particularly in sensitive populations (43). Thus, ethical nudging must be culturally adaptive, yet anchored in universal principles of agency, fairness, and harm prevention.

To support ethical implementation, policymakers and ethics committees should adopt structured evaluation protocols, such as proportionality tests, disclosure standards, equity impact assessments, and sunset clauses for high-influence defaults. Incorporating behavioral ethics into professional training could further strengthen governance and mitigate risks of cognitive paternalism and digital influence asymmetry. Public-sector case studies demonstrate the feasibility of integrating AI-driven behavioral interventions with ethics-by-design principles and participatory oversight frameworks (44). Similar governance challenges have been addressed in domains such as taxation, where AI-powered nudging systems have prompted the development of predictive monitoring tools grounded in ethical compliance protocols (45).

### 4.1 Limitations

This study has several limitations. First, only English-language publications were included, which may introduce linguistic bias and underrepresent relevant scholarship published in Chinese, Japanese, Korean, and other languages—particularly important given the growing ethical discourse in Asian bioethics. Second, gray literature, policy briefs, and government white papers were excluded, potentially omitting pragmatic ethical insights on applied behavioral governance. Third, conceptual and normative studies dominated the dataset, meaning that empirical verification of ethical risks and outcomes remains limited; conclusions are therefore predominantly interpretive. Finally, digital health nudges and AI-mediated behavioral tools were underrepresented in studies before 2020, suggesting that ethical implications of algorithmic nudging require ongoing attention as the field evolves.

Despite these constraints, rigorous screening, independent coding, triangulation, and sensitivity checks strengthened analytic reliability and helped mitigate interpretive bias.

## 5 Conclusion

Healthcare nudges hold substantial promise in advancing public health goals while preserving individual choice. This review demonstrates that ethical legitimacy hinges on transparency, meaningful autonomy preservation, proportionality of influence, and equity in behavioral impact. By grounding analysis in ethical theory and cross-cultural perspectives, our findings contribute to evolving frameworks for responsible behavioral governance in healthcare.

We propose that ethical nudges be governed through transparent disclosure, equity assessment, proportionality justification, and independent oversight. As digital behavioral technologies expand, ongoing empirical evaluation—particularly of patient comprehension, voluntariness, and equity outcomes—will be essential.

Beyond China’s rapidly developing behavioral health environment, these recommendations offer globally applicable insights for ethical policy design, ensuring that nudges function not as covert persuasion tools but as respectful, participatory, and socially equitable public health instruments.

## Data Availability

The original contributions presented in the study are included in the article/supplementary material, further inquiries can be directed to the corresponding author.
